# Efficacy and safety of acupuncture therapy for chronic atrophic gastritis: A meta-analysis and trial sequential analysis protocol

**DOI:** 10.1097/MD.0000000000017003

**Published:** 2019-08-30

**Authors:** Yuan Li, Yili Zhang, Han Meng, Mengting Liao, Zeqi Su, Mengyin Zhai, Lingling Jiang, Ping Li, Xia Ding

**Affiliations:** a Dongzhimen Hospital; b School of Traditional Chinese Medicine; c Beijing Research Institute of Chinese Medicine, Beijing University of Chinese Medicine, Beijing, P.R. China.

**Keywords:** acupuncture, chronic atrophic gastritis, complementary and alternative therapy, evidence-based medicine, traditional Chinese medicine

## Abstract

**Background:**

The proportion of application of acupuncture for chronic atrophic gastritis (CAG) is increasing over time. We will conduct this study to explore the efficacy and safety of acupuncture as a treatment method for CAG.

**Methods:**

We will go through domestic and foreign databases until July 2019 to identify related randomized controlled trials that explored the effectiveness of acupuncture for CAG. RevMan (V.5.3) and test sequential analysis (V.0.9) will be used for mata-analysis and trial sequential analysis.

**Results:**

This study will update previous evidence summaries of acupuncture and determine the efficacy and safety of acupuncture for CAG based on clinical effectiveness rate, clearance of *Helicobacter pylori* (*H pylori*) infection, and quality of life and symptom scores.

**Conclusion:**

This study will determine the evidence for judging whether acupuncture provides benefits in the treatment of CAG, and will support the application of acupuncture in the recovery of patients with CAG.

**Registration number:**

CRD42019127916.

## 1 Introduction

Chronic atrophic gastritis (CAG) is one of the chronic stomach diseases which is defined as the disappearance of the normal glands after suffering repeated damage, with or without intestinal metaplasia.^[1]^ The diagnosis of CAG is mainly based on gastroscopy that is characterized by the visibility of a vascular pattern of gastric mucosa compared with normal mucosa, histopathological examination of gastric mucosa, and pepsinogen I/II ratio.^[2,3]^ Due to the lack of standardized diagnostic criteria, there are few epidemiologic data about its incidence rates, which range from 0% to 10.9% annually around the world according to a meta-analysis of 14 studies.^[4]^ Within the eastern Asian countries of Japan and China, the prevalence rates are higher than other parts of the world and correlate positively with age.^[5,6]^ The clinical symptoms of CAG include both gastrointestinal symptoms, such as stomach distention and stomachache, and non-gastrointestinal symptoms, including anemia and depression. Moreover, CAG is an important precursor lesion in the development of gastric cancer,^[7]^ a major health problem world-wide that ranks fifth for cancer incidence and third for all cancer-related mortality.^[8]^ As a result, it continues to be a significant public health burden while severely impacting humans’ quality of life for those affected.^[9]^ Therefore, sufficient attention should be paid to the management of CAG.

*Helicobacter pylori* (*H pylori*) infection is recognized as the most important cause of CAG, and is also defined as a class I carcinogen of gastric cancer.^[10,11]^ It can trigger a progression from normal mucosa, through chronic non-atrophic gastritis, to atrophic gastritis, then to intestinal metaplasia and dysplasia, and ultimately to carcinoma.^[12]^ However, the management of *H pylori* has now become a challenge. The eradication rate has decreased to below 80%, which is unacceptable in clinical practice and is connected with the increase of antimicrobial resistance, poor compliance, and adverse effects.^[11,13]^ Furthermore, triple and quadruple therapies are crudely equivalent in terms of effectiveness, compliance, and side-effects.^[14]^ Although *H pylori* infection is considered the critical trigger of CAG, a recent meta-analysis has shown that patients receiving long-term treatment with the proton pump inhibitor (PPI) omeprazole have a significantly higher incidence of CAG,^[15]^ as long-term PPI use may change the colonization mode of *H pylori*. This could accelerate the process of gland loss and subsequently result in the appearance of atrophic gastritis.^[16]^ Although the relationship between long-term PPI use and the development of CAG is controversial, this has become a dilemma in decision-making concerning the use of PPIs for maintenance treatment, as it has become one of the most commonly used medications worldwide.^[17]^

At present, there is no effective treatment for CAG. On the basis of receiving *H pylori* eradication therapy, it is important to look for alternative therapies to reduce symptoms of CAG patients and improve their quality of life. Although the proportion of application of acupuncture for CAG is increasing over time, there is no systematic review and meta-analysis of acupuncture on CAG. Furthermore, there are significant differences in the types of acupuncture and the selection of acupoints in the clinical research, and evidence from the usage of acupuncture for CAG treatment in humans is still not sufficiently convincing. This systematic review aims to summarize the existing evidence and evaluate the efficacy and safety of acupuncture as a clinical treatment for CAG.

## 2 Material and methods

This study has been registered at PROSPERO (registration number: CRD42019127916; http://www.crd.york.ac.uk/PROSPERO). We will refer to the preferred reporting items for the systematic review and meta-analysis (PRISMA) to perform this study.^[18]^

### 2.1 Inclusion criteria

#### 2.1.1 Type of studies

We will include all RCTs which explore the specific efficacy and safety of acupuncture in the treatment of CAG. Cross-trials, quasi-RCT, animal experiments and other studies that were repeatedly published or did not have access to complete data will be excluded.

#### 2.1.2 Types of participants

Participants who meet the diagnostic criteria of CAG will be included in this review, regardless of their age, sex, and race.

#### 2.1.3 Types of interventions

We will only include studies which interventions involved acupuncture alone or combined with other western medicine treatments, as well as those with control groups which can verify the therapeutic effect of acupuncture. However, studies that compare the efficacy of different forms of acupuncture will be excluded as this differs from the aim of the review.

#### 2.1.4 Types of comparisons

The control groups which can verify the therapeutic effect of acupuncture will be considered. For instance: acupuncture versus placebo, acupuncture versus no treatment, etc.

#### 2.1.5 Types of outcomes

The primary outcome at the end of treatment or at maximal follow-up is the clinical effectiveness rate, which is categorized as cure, markedly effective, effective, or ineffective according to clinical symptoms, degree of gastric mucosal lesion under gastroscopy and pathological changes of gastric mucosa. The secondary outcomes will include clearance of *H pylori* infection, quality of life (SF-36), symptom scores (stomachache, stomach distention, belching, and acid reflux, etc), and comparison of curative effect of pathological tissue, etc.

### 2.2 Search strategy

We will go through the following 8 databases from inception to July 2019: Cochrane Library, Medline (via PubMed), Web of Science, Clinicaltrials.gov, Chinese National Knowledge Infrastructure (CNKI), Wanfang Data, Chinese Biomedical Literature (CBM), the VIP Chinese Scientific Journal database (CQVIP). “Acupuncture” will be combined with “acupoint,” “traditional Chinese medicine,” “chronic atrophic gastritis,” “randomized controlled trial,” and “CAG” respectively for literature search. The search strategy for selecting the fields of topic, title, or abstract was different referring to the characteristics of databases. The full list of the search strategy for PubMed as follows:

#1. ((((((((((acupuncture [Title/Abstract]) OR needle [Title/Abstract]) OR acupoint [Title/Abstract] OR warm needle [Title/Abstract] electroacupunctur∗ [Title/Abstract] OR electro acupunctur∗ [Title/Abstract] OR fire needle [Title/Abstract] OR flame acupuncture∗ [Title/Abstract] OR auricular needle [Title/Abstract] OR auricular acupuncture∗ [Title/Abstract] OR scalp acupuncture∗ [Title/Abstract] OR head acupuncture∗ [Title/Abstract]) OR traditional Chinese medicine [Title/Abstract].
#2. (((((((Gastritis, Atrophic [MeSH Terms]) OR gastritis [Title/Abstract]) OR chronic atrophic gastritis [MeSH Terms]) OR atrophic gastritis [Title/Abstract]).
#3. (((random [Text Word] OR randomized [Text Word]) OR control [Text Word]) OR controlled [Text Word]) OR trial [Text Word]
#4. #1AND#2AND#3 Filters: Clinical Trial; Humans.
Modified search strategy will be used for other electronic databases.

### 2.3 Selection of studies

**Figure 1:**
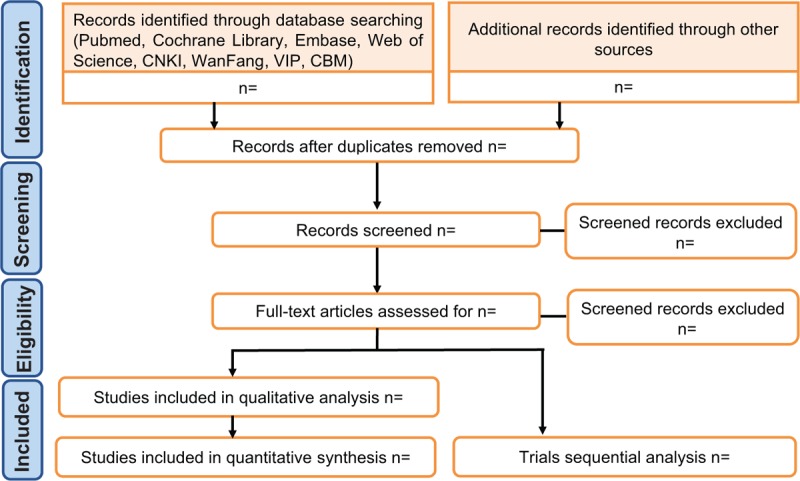
Flow diagram of studies identified.

Search results are imported from the original database into NoteExpress V.3.2.0. Two evaluators (LY and ZYL) will independently evaluate the eligibility of retrieval studies based on inclusion criteria. Titles and abstracts will be reviewed to exclude manifestly inappropriate publications for preliminary research options, and reading the full version is the next step in further assessing the inclusion of the study. The 2 evaluators then will cross-check the selection results. Any differences will be resolved by consensus. Further arguments will be arbitrated by a third commentator (DX). The details of the selection process are shown in Fig. 1

### 2.4 Data extraction

Two reviewers (LY and ZYL) will independently extract details of participants, interventions, comparisons, and results. The following items will be extracted from individual studies: general information, sample size, median age, intervention, acupoints, duration of treatment, and outcomes. If there is any objection, the arbitration will be conducted through discussion or through the third reviewer (DX).

### 2.5 Assessment of risk of bias

The methodological quality of the RCTs will be independently assessed according to the Cochrane Intervention System Review Manual (version 5.1.0). Seven areas will be considered and evaluated 3 levels (“low risk,” “high risk,” or “unclear risk”). The 7 areas include sequence generation, allocation concealment, blindness of participants and personnel, blindness of outcome assessment, incomplete outcome data, selective outcome reporting, and other biases. Two reviewers (MH and LMT) will perfume the evaluation of methodological quality independently, and discrepancies will be solved through mutual consensus (DX).

### 2.6 Data analysis

RevMan 5.3 software (Limited Liability Co., UK) provided by the Cochrane Collaboration will be used for meta-analytic calculations. Statistical heterogeneity was calculated by *I*^2^ test (*I*^2^ <50%) and chi-squared test on Cochrane *Q* statistic, while *P* < .05 or *I*^2^ > 50% was identified as heterogeneous. In the event of significant heterogeneity, the random-effect model was used for estimating the pooled effect size; otherwise, a fixed-effect model was used. If dichotomous, the pooled relative risk with 95% confidence interval will be used as the effect measure. For consecutive outcomes, the mean difference will be used when the units are consistent, and if the outcomes units and/or measurement methods are inconsistent, the standard mean difference will be performed. If the number of included studies is <2 or heterogeneity is apparent, the result of our systematic review will be narratively reported.

### 2.7 Sensitivity analysis and subgroup analysis

If there is significant heterogeneity, the group will be divided into subgroups with similar characteristics according to the characteristics of the study, in order to explore potential sources of heterogeneity. If heterogeneity cannot be resolved (when the *I*^2^ statistic exceeds 50%), no meta-analysis will be performed.

### 2.8 Trial sequential analysis

Trial sequential analysis (TSA) will be conducted to obtain the primary result. Cumulative meta-analysis might result in false positive results (type I error) because of an increased risk of random error from sparse data and repeat significance testing.^[19]^ TSA could control the *P* value and widen the confidence intervals.^[20]^ If the cumulative Z curve entered the futility area or crossed the trial sequential monitoring boundary, the anticipated intervention effect might reach a sufficient level of evidence, and further trials would not be necessary.

### 2.9 Ethics and dissemination

Since this study will not include individuals or animals, ethical approval will not be required. Once the results of the study are obtained, they will be published in conferences or peer-reviewed journals.

## 3 Discussion

To the best of the authors’ knowledge, the present study is the first prospective, randomized, double-blind, controlled trial to investigate the therapeutic effect of acupuncture for CAG. Recent reviews and related experimental studies^[21–23]^ have shown that acupuncture could improve immune function, adjust central neural pathways, regulate gastrointestinal hormones, increase stomach blood flow, regulate cytokines, increase gastric dynamics, control gastric acid secretion, improve inflammatory response, and regulate cell proliferation and apoptosis, which could strengthen the gastric mucosa barrier. Acupuncture is also recommended as one of the treatment methods of CAG in clinical practice.^[24]^ Therefore, it is worth promoting acupuncture in the clinical prevention and treatment of myocardial ischemia with its simplicity, noninvasiveness, and acceptability.

The meta-analysis and trial sequential analysis might be the first to show whether acupuncture therapy has potential benefits for the treatment of CAG. However, before we accept it as an evidence-based treatment option in clinical practice, we need more information to determine the benefit-harm situation. Furthermore, results may be limited by the quality and sample size of English/Chinese articles. Access to new, unpublished data may not be available by the time this review is submitted; therefore, the results presented here may be influenced by publication bias.

## Author contributions

**Data curation:** Yuan Li, Han Meng, Mengting Liao.

**Formal analysis:** Yili Zhang, Ping Li, Yuan Li.

**Methodology:** Yuan Li, Yili Zhang.

**Project administration:** Xia Ding.

**Software:** Mengyin Zhai.

**Supervision:** Zeqi Su.

**Validation:** Xia Ding.

**Visualization:** Yuan Li, Mengyin Zhai.

**Writing – original draft:** Yuan Li, Yili Zhang.

**Writing – review & editing:** Lingling Jiang.
