# Efficacy and safety of acupuncture therapy for chronic atrophic gastritis: A meta-analysis and trial sequential analysis protocol: Erratum

**DOI:** 10.1097/MD.0000000000017514

**Published:** 2019-10-04

In the article, “Efficacy and safety of acupuncture therapy for chronic atrophic gastritis: A meta-analysis and trial sequential analysis protocol”,^[1]^ which appears in Volume 98, Issue 35 of *Medicine*, “YLZ, YZ, HM, and MTL have contributed equally to this work” in the footnote should appear as “YL, YLZ, HM, and MTL have contributed equally to this work.”
